# An Automatic Critical Care Urine Meter

**DOI:** 10.3390/s121013109

**Published:** 2012-09-26

**Authors:** Abraham Otero, Roemi Fernández, Andrey Apalkov, Manuel Armada

**Affiliations:** 1 Department of Information and Communications Systems Engineering, University San Pablo CEU, Boadilla del Monte, 28668 Madrid, Spain; 2 Centre for Automation and Robotics, CSIC-UPM, Ctra. Campo Real, Km. 0,200, La Poveda, Arganda del Rey, 28500 Madrid, Spain; E-Mails: roemi.fernandez@car.upm-csic.es (R.F.); apalkov.andrey@gmail.com (A.A.); manuel.armada@csic.es (M.A.)

**Keywords:** biosensors, urine output, critical care, capacitive sensors, patient monitoring

## Abstract

Nowadays patients admitted to critical care units have most of their physiological parameters measured automatically by sophisticated commercial monitoring devices. More often than not, these devices supervise whether the values of the parameters they measure lie within a pre-established range, and issue warning of deviations from this range by triggering alarms. The automation of measuring and supervising tasks not only discharges the healthcare staff of a considerable workload but also avoids human errors in these repetitive and monotonous tasks. Arguably, the most relevant physiological parameter that is still measured and supervised manually by critical care unit staff is urine output (UO). In this paper we present a patent-pending device that provides continuous and accurate measurements of patient's UO. The device uses capacitive sensors to take continuous measurements of the height of the column of liquid accumulated in two chambers that make up a plastic container. The first chamber, where the urine inputs, has a small volume. Once it has been filled it overflows into a second bigger chamber. The first chamber provides accurate UO measures of patients whose UO has to be closely supervised, while the second one avoids the need for frequent interventions by the nursing staff to empty the container.

## 1. Introduction

Nearly any physiological parameter of a patient admitted to a critical care unit can be (and often is) sensed automatically by sophisticated commercial monitoring devices and displayed on a screen next to the patient's bed. This provides invaluable information for interpreting the patient's state. In most cases, these devices also provide supervision capabilities that check if the parameters' values remain within a pre-established range considered as normal for each patient [1]. This range represents the values considered as physiologically acceptable for each parameter. If a parameter does not fall within its acceptable range, audible warnings to alert the health care staff are generated [2].

These monitoring devices discharge the healthcare staff of a considerable workload, since they eliminate the need to continuously supervise the values of the physiological parameters of every patient. Furthermore, they also avoid human errors, which are common in any repetitive and monotonous task such as the supervision of physiological parameters [3,4].

Arguably, urine output (UO) is the most relevant physiological parameter which still is measured and supervised manually by healthcare staff. UO is the best indicator of the state of the patient's kidneys. If an adequate amount of urine is being produced, the kidneys are well perfused and oxygenated. Otherwise, the patient is suffering from some complication [5]. The measurement of UO is necessary for calculating the patient's water balance; and UO is used in multiple therapeutic protocols to assess how the patient reacts to the treatment. Two therapeutic protocols where this parameter plays a central role are the resuscitation and early management of burn patients [6] and the resuscitation of septic shock patients [7]. In the case of the latter, achieving a certain minimum value for the UO itself is a therapeutic goal.

When the UO is too low, the patient is said to have oliguria. If the patient does not produce urine at all, then he/she is said to have anuria. The most common causes of oliguria and anuria are (1) prerenal azotemia, which can be the result of infectious diseases and/or heart failure, (2) postrenal azotemia by obstruction of the urine flow, which can be caused by an enlarged prostate, compression of the urethra by a tumor, an expanding hematoma or fluid collection, and (3) intrinsic kidney damage, which can be caused by acute tubular necrosis, rhabdomyolysis, medication and/or glomerulonephritis [5]. When the UO of a patient is too high, the patient is said to have polyuria. Diabetes is the most common cause of polyuria [8].

Currently, the urine of critical care patients is collected in a graduated container that is connected to the patient's bladder through a Foley catheter. Measurement is carried out manually by nursing staff that get the reading from the graduated container. This task is significantly time-consuming. To take a measure, a nurse must put on gloves since he/she is going to manipulate body fluids, walk to the patient's bed, take the measure visually, write it down in the nursing worksheet, operate a valve which releases the urine of the graduated container into a plastic bag, wait for the urine to drain, close the valve, check that the valve is properly sealed and check if the plastic bag connected to the graduated container needs to be emptied. This procedure can take approximately 2 minutes per measurement. In critical care units of first world countries, measurements of every patient's UO are taken hourly, 24 times a day, 365 days a year. If there are 15 patients in the unit, this means 30 minutes per hour; 12 hours a day. In emerging countries, the more limited availability of healthcare staff often prevents them from spending this amount of time on this task. In their case, often only burn patients—for whom UO monitoring is of paramount importance—have this parameter recorded every hour, while the remaining critical patients have it recorded every 2 or 3 hours.

It can even be argued that the recording interval currently employed in first world countries—once every hour—also is a compromise between avoiding risk states for the patient and relieving the nursing staff of an excessive burden. Hence, there is interest in developing a device capable of automatically monitoring UO. Such a device would decrease the workload associated with this task and, at the same time, it would permit supervision to take place on a more continuous basis.

This paper presents a patent-pending device capable of measuring continuously the patient's UO [9]. The device also enables automatic supervision of UO therapeutic goals. In Section II of the paper we review related work. Section III covers the design of the device and Section IV its validation. Finally, the results of this work are discussed, and a series of conclusions are given. This article is an extension of the conference paper [10].

## 2. Related Work

The automation of UO measurement has only begun to be addressed in the literature in the last three years. Common industrial solutions to measuring the height of a liquid column inside a container are not suitable for this task. Automatic UO measurement requires a device capable of measuring very small amounts of liquid flow (as low as 3–5 milliliters per hour). This precludes the use of cheap ultrasound-based solutions [11] or of commercial flow meters because they lack the required accuracy. On the other hand, any component of the device that is in contact, or may be in contact, with the urine cannot be reused on other patients and must be replaced approximately every 4–7 days for hygienic reasons. This precludes the use of expensive high accuracy laser-based solutions [12] that are not cost effective.

The first device to automatically measure the UO of critical care patients dates from 2009, when the Israeli company Medynamix developed a device, Urinfo 2000, that measures UO by counting the number of drops of urine produced by the patient [13]. The device was designed to automate the hourly UO measurement, but not to take more frequent measures. Its accuracy is 8% (±25 mL) [13]. At present, Medynamix has been bought by Flowsense, a construction services company that is starting to market the device [14].

That same year we developed a device to measure UO automatically [15]. It is based on the use of a high precision scale that measures the weight of a commercial urine meter. A support frame made up of Bosch profiles located on the scale's pan isolates the scale from force transmission from the patient's bed and guarantees a continuous and smooth flow of the liquid through the urine meter's input tube. The error in the device's measures is below 1.5%. This device was designed with the goal of conducting a series of clinical studies based on more continuous and accurate monitoring of UO. It is currently being used at a research unit associated with the University Hospital of Getafe, in Spain, to study sepsis in an animal model. This pathology usually causes renal failure, hence the interest in accurately monitoring renal function [16].

The scale-based device has high accuracy and acquisition rate, which make it ideal for carrying out clinical studies. However, its size and operation make it tedious for use in a critical care unit. Its high cost is also a weak point. We tried to design a device that would address these problems. The second device uses reed switches activated by a magnetic float to measure the amount of urine collected in two containers [17]. An electronic unit calculates the urine produced by monitoring the state of the reed switches. Although from a technical point of view this solution is sound and solves the problem, it has a practical problem: use in the clinical routine requires a partnership with a hospital supplier company. The ideal partner would be a company that is already supplying hospitals with manual urine meters. However, these companies are reluctant to build a new urine meter from scratch. This would require new manufacturing processes and machinery. Probably, during a transition period it would be necessary to manufacture both the old and the new models. Moreover, the intellectual property rights that may be protecting their current manual urine meter may no longer be useful to protect the new device from competitors. Building a new device from scratch that provides similar functionality to that of products that are currently marketed has considerable disadvantages and risks. For a company, adding something that provides the new functionality to their current device is a much more attractive scenario. All the machinery and current manufacturing processes remain unchanged. The new add-on can be manufactured separately and installed on the units that will be sold with the new features. All the intellectual property rights that may be protecting the old device will continue to protect the new one.

The goal of this paper is to build a device that is suitable for use in the clinical routine that can be manufactured as an add-on to commercial manual urine meters.

## 3. Materials and Methods

### 3.1. Capacitive Sensors

**Figure 1.**
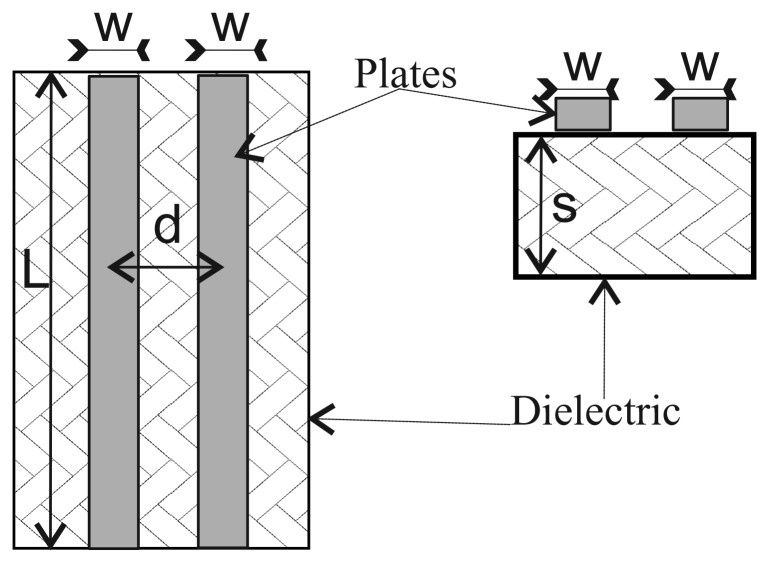
Coplanar-plate capacitor.

**Figure 2.**
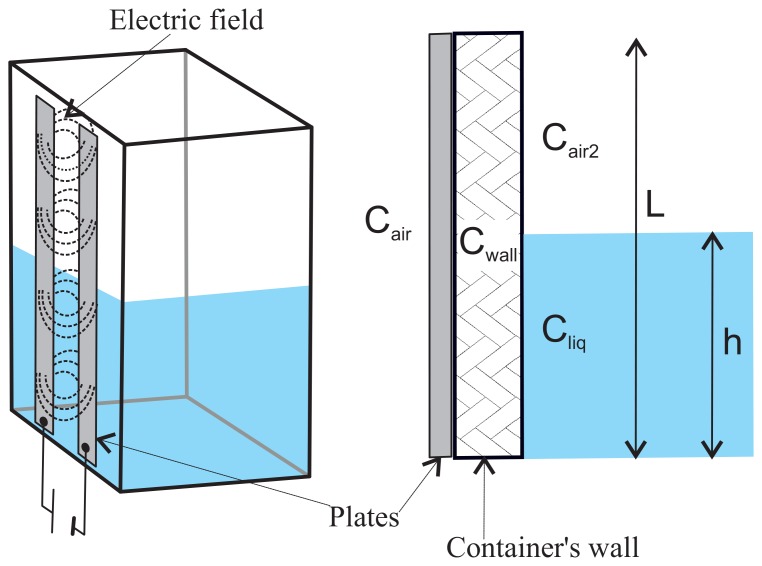
Use of a coplanar plate capacitive sensor for measuring the height of the column of liquid in the container. On the left, the location of the capacitor's blades on the container is shown. On the right, the different capacities that come into play are shown.

It is possible to measure the height of the column of liquid accumulated in a container made of dielectric material from the exterior by using a capacitive sensor [18]. A common type of capacitive sensor is based on a coplanar-plate capacitor [19]. These sensors are made up of two elongated conductive blades placed in parallel, at a distance *d* of each other (see Figure 1). In our case, the length of the blades must be at least equal to the maximum height of the column of liquid to be measured. The blades should be placed lengthwise along the container's vertical dimension (see Figure 2).

If we have a dielectric material with dielectric constant ∈ on one of the sides of the plates of a coplanar-plate capacitor, the capacitance due to the presence of the dielectric material is given by [20]:

(1) $$C=L\;\in \;\frac{K(k\prime )}{2K(k)}$$

where *L* is the length of the plates, and *K*(*k*′) and *K*(*k*) are complete elliptic integrals of the first kind. $k\prime =\sqrt{1-k^{2}}$, and

(2) $$k=\frac{\text{tanh}\left(\frac{\pi d}{4s}\right)}{\text{tanh}\left(\frac{\pi (w+d/2)}{2s}\right)}$$

where *d* is the distance between the two plates, *w* is the width of the plates, and *s* is the thickness of the dielectric material (see Figure 1). If *s* = ∞, then Equation (2) can be simplified to *k* = *d*/(*w* + *d*/2).

For the sake of simplicity, we shall rewrite Equation (1) as:

(3) C=L∈f(d,w,s)

where f has no dependence with the length of the plates, or with the dielectric constant of the material.

**Figure 3.**
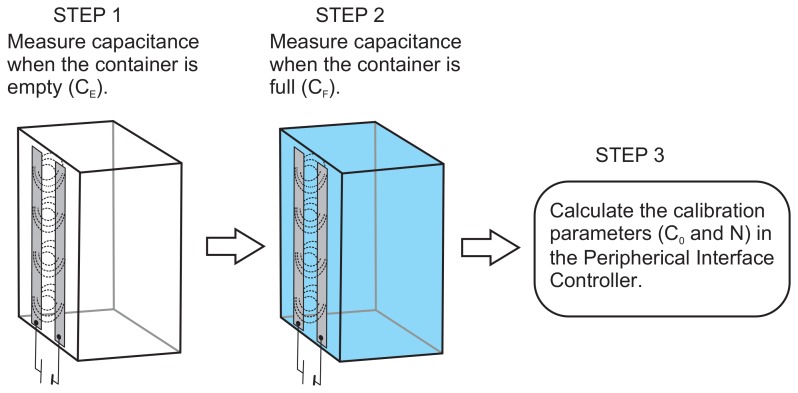
Diagram of the calibration process.

In our problem, on one side of the capacitor, the dielectric will be the air. On the other side, we first have the wall of the container, and then air, or liquid, or a combination of both (see Figure 2). In this situation, it can be proved that the total capacitance of a multi-dielectric layer coplanar-plate capacitor can be calculated as the sum of four terms, each of them given by Equation (1) [21]:

(4) $$C_{s}=C_{\text{air}}+C_{\text{wall}}+C_{\text{liq}}+C_{\text{air}2}$$

where *C_air_* is the capacitance due to the presence of air outside the container, *C_wall_* is the capacitance due to the container's wall, *C_liq_* is the capacitance due to the height of the liquid column inside the container, and *C_air2_* is the capacitance due to the presence of air in the upper part of the container (see Figure 2). To calculate *C_air_*, *C_liq_* and *C_air2_* the thickness of the dielectric material can be considered to be infinite. However, to use the capacitive sensor, we need not to calculate the precise values of these four terms. Equation (4) can be rewritten as:

(5) $$C_{s}=L\;\in_{\text{air}}f(d,w,\infty )+L\;\in_{\text{wall}}f(d,w,s_{\text{wall}})+h\;\in_{\text{liq}}f(d,w,\infty )+(L-h)\;\in_{\text{air}2}f(d,w,\infty )$$

where *h* is the height of the liquid column in the container and *L* is the height of the container. In our system, all parameters of Equation (5) but *h* are constant. Therefore Equation (5) can be rewritten as:

(6) $$C_{s}=C_{0}+(\in_{\text{liq}}f(d,w,\infty )-\in_{\text{air}}f(d,w,\infty ))h=C_{0}+Nh$$

where both *C*_0_ and *N* are constants that can be calculated empirically from the formulas presented here, but they can also be calculated experimentally in a simple manner (see Figure 3). To this end, we shall measure the capacitance of the sensor when there is no liquid in the container; *i.e.*, *h* = 0. We shall call this capacitance *C_E_*. From Equation (6) we obtain that *C*_0_ = *C_E_*.

Then, we shall measure the capacitance when the container is completely full. Let us suppose that the measured capacitance is *C_F_*. If the height of the liquid column at this moment is *L* then:

(7) $$C_{F}=C_{E}+NL\Rightarrow N=\frac{C_{F}-C_{E}}{L}$$

With this calibration process we avoid the need to know the precise values of the dielectric constant of the walls of the container, of the liquid, the width of the container's wall, or any other geometric feature of the device.

After the calibration, if we measure the capacitance of the capacitor at any instant *t*, *C_t_*, we can calculate the height *h_t_* of the liquid column in the container at *t* as:

(8) $$h_{t}=\frac{\left(C_{t}-C_{E}\right)}{\left(C_{F}-C_{E}\right)}L$$

where *C_E_* and *C_F_* were measured in the calibration process. From *h_t_* we can calculate the volume of liquid in the container if we know its area.

**Figure 4.**
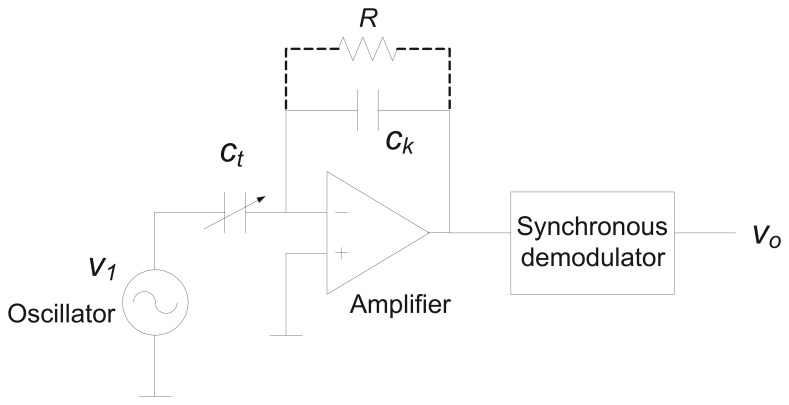
Measurement of the capacitance of the capacitive sensor.

A signal conditioning circuit can be used to measure the capacitance *C_t_* by converting capacitance variations into a voltage modulation. Since the capacitive sensor is a passive component, an excitation signal is required for the measurement. A high-oscillation frequency nearby the resonance frequency of the circuit is preferred for the excitation in order to achieve a low electrode plate impedance and to maximize the sensitivity to the height of the column of liquid. The excitation wave usually has a square or trapezoidal shape. A triangular waveform can also be used to allow a simpler amplifier with resistive feedback. However, a sine wave offers better accuracy at high frequency. Then, the signal is amplified, demodulated and filtered for further processing and A/D conversion. Sensors excited by a continuous wave signal usually use synchronous demodulators, which offer high precision and good rejection of out-of-band interference [22]. The synchronous demodulator behaves essentially as a double wave rectifier, making this correction in sync with the excitement signal or synchronism of the circuit. During the first half of the cycle, the excitation signal value is superior to a reference, and the demodulator works as a rectifier inverter. During the second half of the cycle, the excitation signal is lower than the reference and the demodulator works as a follower of tension, so that the entrance to the demodulator appears in the output with a unit gain. Finally, a low pass filter removes the frequency of the carrier and other harmonics of higher-order, producing a ripple-free output. The amplitude of the rectified and filtered signal is related to the magnitude of the height of the column of liquid, while the polarity indicates the sense of displacement. Thus, with the configuration shown in Figure 4, the height of the column of liquid *h_t_* is given by:

(9) $$h_{t}=\frac{L}{\left(C_{F}-C_{E}\right)}\cdot (C_{k}\left|\frac{v_{0}}{v_{1}}\right|-C_{E})$$

where $\left|\frac{v_{0}}{v_{1}}\right|$ is the gain of the AC amplifier, and *C_k_* is a constant capacitance in the feedback loop of the AC amplifier. A resistance R in Figure 4 may be used to control the DC bias on the AC amplifier.

### 3.2. The Container

An automatic urine meter must at least be capable of providing accurate measurements of urine volumes as small as 3–5 milliliters per hour. A capacitive sensor measures the height of the liquid column. The error in the measurement of the height will grow proportionately with the area of the container when calculating the volume of liquid. Thus, to accurately measure small volumes of liquid, the area of the container where the liquid accumulates must be small.

Current clinical protocols specify that UO should be recorded every hour; *i.e.*, the nursing staff manually operates the valve which releases the urine of the container only once every hour. Therefore, the container must be able to store the UO that can be produced in one hour. If the patient is producing normal amounts of urine, or worse, if the patient has polyuria, the patient could produce up to 500 mL of urine per hour. If the graduated container has a small area, it would need to have a disproportionate height to collect all the urine produced over an hour. In practice, it is not viable to use such small containers with the necessary height.

The most common solution for this problem, and the one used by all the companies that manufacture manual urine meters, is to use two containers connected to each other. The patient's urine flows into a small container—usually from 35 to 50 mL in commercial urine meters—with a small area base. If the patient has oliguria or anuria, his/her hourly UO will be lower than the volume of this container. This small container enables accurate UO measurement where high accuracy is required—when the patient has oliguria or anuria. This small volume container can overflow if the patient produces normal amounts of urine, and if the patient has polyuria it will overflow. However, in these situations it is not important to have a highly accurate measurement. The small container overflows into a larger volume container—usually from 350 to 500 mL in commercial urine meters. This enables less accurate UO measurement of large UO volumes where high accuracy is not required.

**Figure 5.**
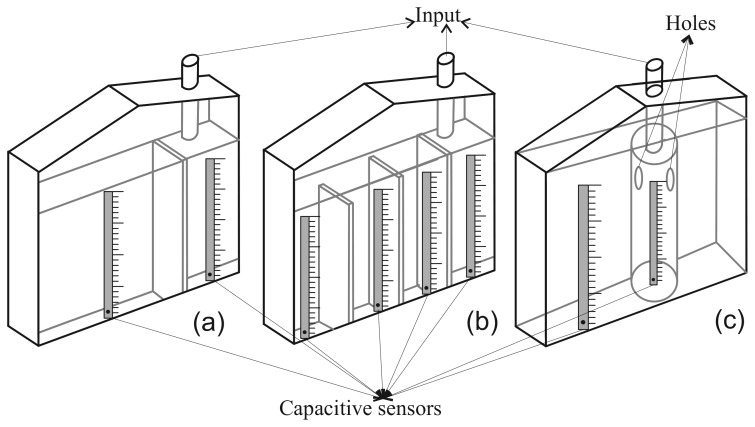
Different designs for the graduated containers of commercial urine meters and possible placement positions of capacitive sensors.

In order for the small container to overflow into the large one, a single parallelepiped container can be divided with a vertical wall with a height less than the height of the parallelepiped. The vertical wall must be closer to one end of the parallelepiped container than to the other so it produces chambers with different volumes (see Figure 5(a). Urine is collected initially in the smaller chamber, and it can overflow into the larger one. Some commercial urine meters divide the parallelepiped container into several chambers that overflow consecutively into each other (see Figure 5(b). A third solution is to have a graduated container of small volume inside a larger volume graduated container (see Figure 5(c). The small container has holes in its upper part, so that when the urine reaches the level of the holes it overflows into the large chamber (see Figure 5(c).

All models of commercial urine meters we know about follow one of these three designs. In all cases the containers are made of transparent plastic and are provided with a graduated scale for visual measurements of the volume of urine contained in each chamber. The container is equipped with a valve that releases the contents of all chambers into a plastic bag. These bags usually have a volume ranging from 1,500 to 2,500 mL. When the bag is full, it is replaced by the healthcare staff.

**Figure 6.**
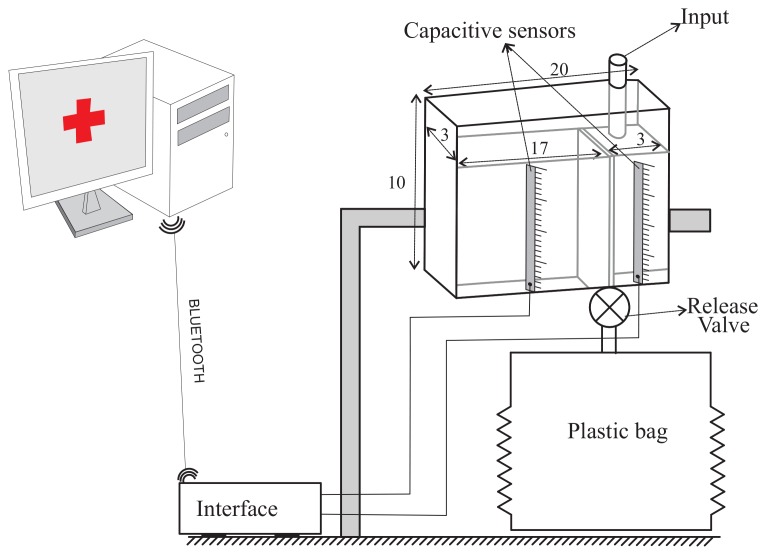
Diagram of the device.

To sense the UO we will use a capacitive sensor to measure the height of the column of liquid of each chamber of the container (see Figure 6). In all cases, the capacitive sensors are located in the outside of the container. Therefore, they do not come into contact with the urine and there is no reason to discard them when the urine meter is replaced. In the patent application for the device [9] several mechanisms to fix the sensors to the container wall are proposed. One solution is to build slots or sheaths in the wall of the container. The longitudinal blade of the capacitive sensors would be inserted into them. If the goal is not to make any changes to the container (because, for example, we are using a commercial urine meter as a basis to build our automatic urine meter), an adjustable frame made of telescopic slides, rubber bands or even duck tape can be used to fix the sensors. Either of these solutions should make it easy to install and remove the sensors, so that they can be reused.

### 3.3. Our Prototype

We have built a prototype of the device using capacitive sensors manufactured by the Sensortechnics GmbH and a transparent plastic container following the design shown in Figure 5(a). The container has a dimension of 20 × 3 × 10 cm^3^. and its wall thickness is 3 mm. The container is divided into two chambers. The small one (3 × 3 × 8 cm^3^) is separated from the large one (15 × 3 × 10 cm^3^) by a wall of 8 cm in height. The physical features of the container are similar to those of the graduated containers of commercial urine meters (see Figure 6).

Each capacitive sensor includes an integrated conditioning circuit for providing an analog output ranging from 0.5 V to 4.5 V and that is linearly proportional to the height of the column of liquid collected in the chamber of the container (see Equation (9)). An interface circuit was built to enable communication between the sensors and a serial port to Bluetooth adapter, which sends the readings to and receives the commands from the central PC. This circuit is based on a Peripherical Interface Controller that has an Addressable USART module, multiple interrupt sources and an Analog to Digital Converter module with 8 input channels. Therefore, up to a maximum of 8 capacitive sensors can be connected to this interface circuit. The A/D module allows conversion of each analog output signal *v*_0_ from the capacitive sensors to a corresponding 10-bit digital number, which means an A/D voltage resolution of 4.9 mV/bit for a full scale measurement range of 0 V to 5 V. This resolution is more than enough if we take into consideration that the typical sensitivity of the selected sensor is 40 mV/mm. After the A/D conversion, Equation 9 is locally computed in the Peripherical Interface Controller using the previously stored values of *C_E_*, *C_F_*, *C_k_*, *L* and *v*_1_. The interface device also has an interrupt priority feature that allows each interrupt source to be assigned a high priority level or a low priority level. The USART is configured as a full duplex asynchronous system.

The UO readings are then acquired every 5 seconds by a Java program running on the central PC. The hits and shakes caused by healthcare staff when they manipulate the patient, or by the patient himself, can produce movements of liquid in the graduated container, and therefore result in artifacts in the sensors' readings. To remove these possible artifacts, the UO value for each minute is the median of the 12 samples taken during that minute.

A Java application running on a PC displays a graph showing the patient's UO on a minute by minute basis. It also allows the healthcare staff to set the therapeutic goals for UO by indicating the range of acceptable values. If UO is less than the minimum acceptable, the patient has oliguria or anuria. If it is more than the maximum acceptable, the patient has polyuria. If the readings for a consecutive five-minute interval lie outside the range of ideal values, the program produces an audible warning until it is turned off by the healthcare staff. The length of this interval can be configured from the application.

One of our goals is to reduce the time that the healthcare staff needs for tasks related to UO supervision. Despite the fact that the healthcare staff does not need to take visual measurements of UO, they still need to walk to the patient's bed every hour to open the valve that releases the urine of the graduated container into the plastic bag. The Java application alerts healthcare staff when to empty the graduated container in order to minimize the number of visits to the patient's room. As long as the patient is within the therapeutic goals, we shall not alert the healthcare staff to empty the graduated container until its small chamber is full, and the larger chamber is filled up to 95% of its capacitance. If therapeutic goals are not being met but the patient has polyuria, we shall act the same way as in the previous case. In these situations there is no need for accurate supervision of UO. If the patient has oliguria or anuria we shall ask the healthcare staff to empty the graduated container when its small chamber is filled to a level of 95% of its capacitance. In this scenario we should only use measurements from the small chamber, given that the patient's UO needs to be monitored accurately.

## 4. Prototype Testing

**Figure 7.**
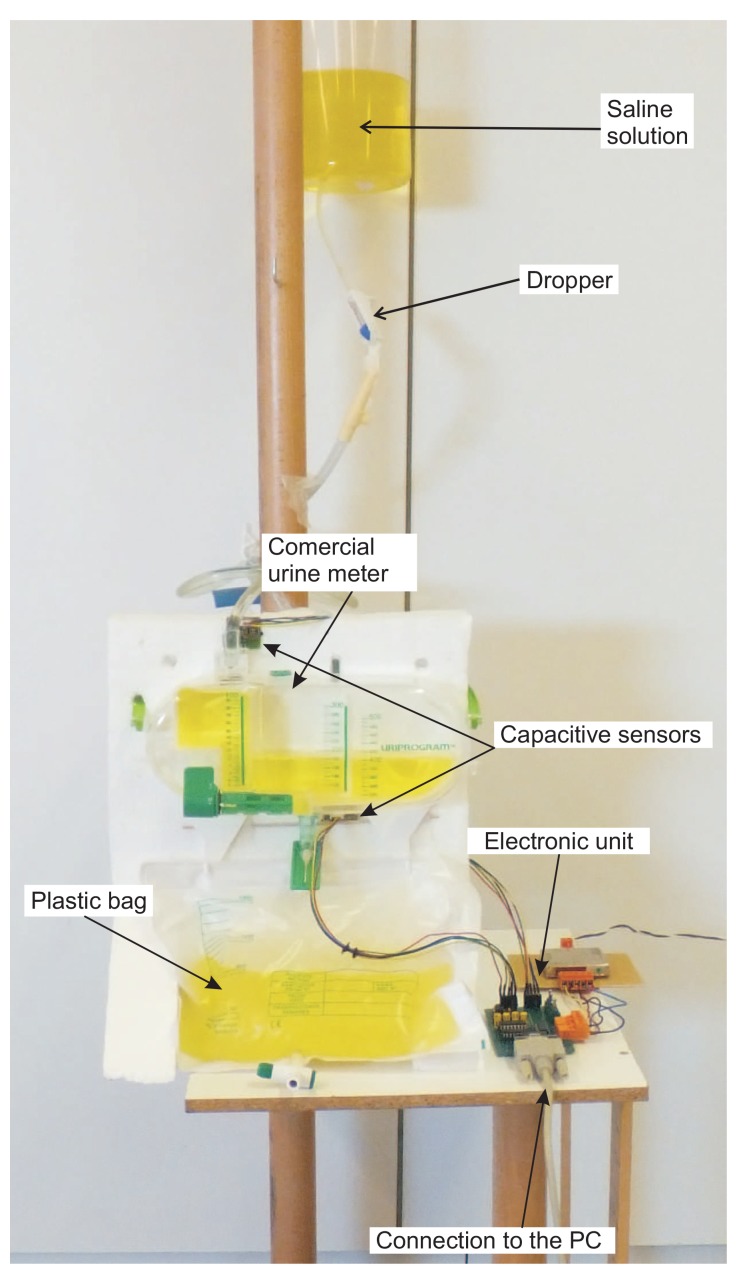
Prototype of our device built with a commercial urine meter.

We have verified the proper operation of the prototype device in a series of tests. In the tests, a saline solution with similar properties to urine was used. This liquid was stored in a container and a dropper was used to simulate different flow rates (see Figure 7). The graduated container, with the two capacitive sensors, was placed on the plate of a high-precision industrial scale—a PGW 4502e, built by Adam Equipment Inc. The capacitive sensors were connected to a PC. The PGW 4502e scale is equipped with its own serial port that permits querying for readings. We built a program that periodically takes measurements from the scale. This program was running on a second PC which was connected through the serial port with the scale. Given that the density of the saline solution was known, we can determine the volume of liquid in the container at any time by subtracting from the weight measured by the scale the weight of the empty container, and dividing the result by the saline solution density.

This set up allows us to automate the process of carrying out multiple measures of the volume of liquid calculated both from the measurements of the capacitive sensors and from the weight of the scale. The PGW 4502e scale has an accuracy guaranteed by the manufacturer of 0.01 g. Therefore, we shall consider that measures of the volume of liquid obtained from the scale are the ground truth against which we shall compare the measurements obtained from the two capacitive sensors.

Five different rates of urine production were simulated using the dropper: approximately 200, 400, 1,000, 2,000, and 3,000 mL/day. Although the precise interpretation of these values depends on the patient's condition and weight, usually 200 mL of urine per day correspond to severe oliguria, 400 mL/day corresponds to moderate oliguria, 1,000 and 2,000 mL/day are normal/healthy amounts, and 3,000 mL/day corresponds to polyuria. For each urine production rate, four different experiments were carried out, each with a duration of 4 hours. Simultaneous minute by minute liquid volume measurements were taken using the scale and the capacitive sensors.

Table 1 summarizes the results of the tests for each of the flow rates. The first column shows the root-mean-square error (RMSE) of the minute by minute measurements obtained from the sensors compared to measurements obtained from the scale during the four hours. The next four columns show the error of the measurement in the minutes 60, 120, 180 and 240 as a percentage of the total UO produced up to that moment. In all cases we show the mean ± standard deviation of the four experiments carried out for each flow rate. The values marked with “*” correspond to measurements in which only the small chamber of the container had liquid (it had not yet spilled into the larger chamber). The RMSE of these measures is smaller; the same error in the calculation of the height of the liquid column will yield a smaller error in the volume calculation if the calculation is made over the small chamber, and will lead to a larger error if the calculation is made over the big chamber.

The density of human urine can vary between 1.005 and 1.035 g/mL [5]. It has an average value of approximately 1.020 g/mL (this was the density of the saline solution used in the tests summarized in Table 1). These variations in density are due to the urine containing different concentrations of various components such as urea, chlorine and sodium. Sodium is usually the most abundant in urine and its concentration also tends to vary more significantly—from 40 to 240 mEq/L. An increase or decrease in sodium concentration can change the dielectric properties of urine. However, in Equation (6) we assumed that the dielectric properties of urine were constant.

**Figure 8.**
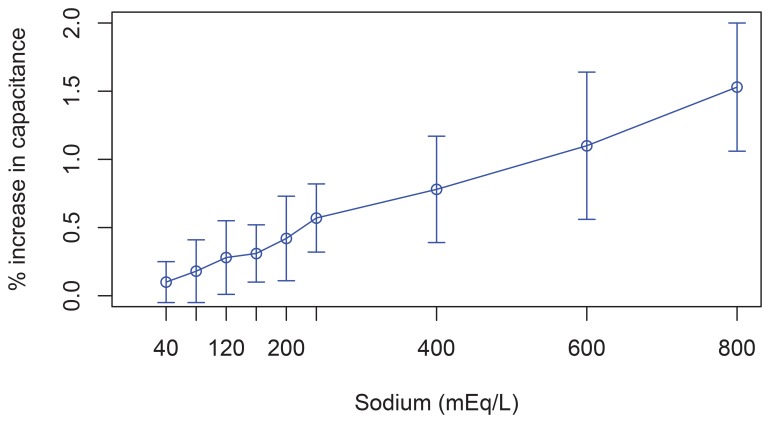
Change in the capacitance when changing the sodium concentration. The last three concentrations are above the physiological thresholds of this parameter for human beings.

We have conducted a series of experiments to quantify the effects of the variations in the dielectric properties of urine. We placed a capacitive sensor on the outside wall of a container filled with distilled water (≃0 mEq/L of sodium) and we measured the capacitance of the capacitor. We then added sodium chloride to the container and waited for it to dilute. We measured again the capacitance to assess how the capacitance was affected by the presence of sodium. Experiments were performed with concentrations of sodium of 40, 80, 120, 160, 200, 240, 400, 600 and 800 mEq/L. The last three concentrations are above the physiological thresholds of this parameter for human beings. For each concentration, the experiment was repeated four times. Figure 8 shows the increase in the capacitance (mean ± sd) as the percentage of increase with respect to the capacitance measured with the distilled water. The maximum variation in capacitance if the concentration is maintained within the physiological limits of human beings (240 mEq/L) was 0.57 ± 0.16% compared to distilled water and 0.46 ± 0.15% compared to the lowest concentration within physiological limits (40 mEq/L). The variation in capacitance for 800 mEq/L was 1.53 ± 0.23%.

Although these tests were conducted with a container completely filled with liquid, as can be seen in Equation (6), for any height of the column of liquid a change in the concentration of sodium (and therefore on the dielectric properties of the liquid) will produce the same change in percentage in the measure of the capacitance for any height of the liquid column.

## 5. Discussion

The measurement error of the device in all the test conditions is lower than the Urinfo 2000 mean error (8% [13]). The device accuracy also compares favorably with the accuracy of the visual measurements of the nursing staff, whose average error has been reported to be as high as 26% [13]. However, the accuracy is considerably lower than the device previously developed by the authors [15]. Compared with the scale-based solution, this device has the advantages of being smaller and easier to use and having a lower cost.

The impact of changes in the sodium concentration in urine on the accuracy of the device is under 1% for variations within the physiological thresholds of this parameter for human beings. The ambient temperature may also impact the dielectrics properties of both the urine and the container's wall. However, in an ICU temperature is controlled by air conditioning systems to ensure that the temperature will always be approximately constant and optimal for the recovery of patients. Thus, variations in temperature are minimal and should not have an appreciable impact on the accuracy of our device.

All of the devices previously built to automate the measurement of UO are solutions that have to be built from scratch. The device presented in this paper can be used as an add-on for commercial urine meters to transform these manual measuring devices into automated measuring devices without requiring modifications. They all use rigid plastic containers with two or more independent chambers following one of the three designs shown in Figure 5. Therefore, an adjustable frame made of telescopic slides, rubber bands or even duck tape can be used to fix the capacitive sensors to their containers.

This feature is advantageous for taking the device to the clinical routine. From the companies point of view, building a new device from scratch that provides similar functionality to that of one of its products has considerable disadvantages and risks. At least during a transition period, it would be necessary to continue manufacturing both the old and the new devices; *i.e.*, the manufacturing infrastructure would have to be duplicated and the logistics would be complicated. On the other hand, companies often have multiple patents to defend their urine meters from being copied by competitors. Using a completely new device, even if it was protected by a patent, could weaken the company's position in the market. For companies, adding something that provides new functionality to their current device is a much more attractive scenario. In this way, all the machinery and current manufacturing processes remain unchanged. The new add-on is manufactured separately and installed on the units that will be sold with the new features. All the patents that protected the old device will continue to protect the new one, plus the patent or patents related to the new addition. The company's customers can make a gradual transition from the old device to the new one.

From the health care staff point of view, it is easier to learn to operate a device that is a slight variation of an existing one than a completely new device. Furthermore, our solution allows the health care staff to continue taking visual measurements of UO in parallel with the automatic measurements. This can be very useful for a transition period in which they learn to use and trust the new system, and they will be less reluctant to accept the new system if they know that at any time they can return to the previous solution.

Measuring UO automatically enables automatic supervision of the therapeutic goals. It can also be used to alert caregivers whenever it is necessary to open the valve that releases the urine from the graduated container to the plastic bag, minimizing the number of visits to the patient's bed for tasks related to monitoring UO. The overall result is a significant reduction in the healthcare staff workload. As we argued in the introduction of the article, in a critical care unit of a developed country with 15 patients, up to 12 hours per day are necessary for tasks related to supervising UO. Typically, nurses work in shifts of six hours. Therefore, each day two complete nursing shifts are required for these tasks. Not needing to take manual measures, having automatic supervision, and reducing the number of visits to the patient's bed results in a considerable reduction in the nursing staff workload associated with the monitoring of UO. Therefore, automating UO supervision could translate into cost savings for institutions that provide health services.

Furthermore, our device provides feedback on the status of the patient's kidneys more frequently than is currently available in critical care units—hourly. This could allow the clinician to react more promptly to complications in the state of the patient. Therefore, the device has the potential of improving patient outcomes. There are already authors supporting this point of view [23,24], and our own studies on the minute by minute measurement of UO of septic pigs supports this thesis.

No components of our solution are in contact with the patient's urine. They can be installed and removed easily by the healthcare staff. Thus, they need not be discarded when the urine meter's container needs to be discarded. The components suffer no significant degradation caused by operation either. Therefore, the cost of the system (approximately $400 for each patient bed, plus a single central PC) can be amortized over a long period of time and their economic impact on the institutions that provide the healthcare services is negligible.

Finally, it should be noted that the calibration process probably does not need not be performed for each unit that is manufactured. As long as the shape, size and material of the container remain unchanged, and the sensors' position is the same, all units should have the same calibration parameters. Proper placement of the sensors can be ensured by building slots or sheaths in the wall of the container to enclose the sensors, or by providing reference marks to indicate the position where the sensors should be installed. If the manufacturing process can ensure that the device's parameters have tolerances within acceptable ranges, per device calibration should not be required. Instead, the same values could be used for all of them.

## 6. Conclusions

We have presented a device that provides continuous and accurate measurements of patient's UO. Two capacitive sensors take continuous measurements of the height of the liquid column accumulated in the two chambers of a container. Urine enters the container through the first chamber, which has a small volume. Once it has been filled, it overflows into a second bigger chamber. The first chamber provides accurate UO measures, while the second one avoids the need for frequent interventions by the nursing staff to empty the container. The measures are sent via Bluetooth to a Java program running on a PC, which calculates the UO from this information and supervises the achievement of therapeutic goals.

The automation of the tasks of measuring and supervising UO results in a reduction in the healthcare staff workload. At the same time, the device provides feedback on the status of the patient more frequently than is currently available. This could result in patient outcome improvement. Finally, the cost of the solution for the institutions that provide health services is negligible. The device presented in this article adds non-disposable parts to the design of current commercial urine meters. Given that these parts are not disposable, their cost (approximately 400 $ for each patient bed) can be amortized over a long period of time.

## Figures and Tables

**Table 1.** Validation results for the different flow rates. Values marked with “*” correspond to measurements involving only the small chamber of the container.

Flow rate	RMSE	% error at 1 hour	% error at 2 hour	% error at 3 hour	% error at 4 hour
200	0.63 ± 0.092*	6.25% ± 1.38*	3.53% ± 0.98*	2.76% ± 0.87*	2.05% ± 0.76*
400	0.76 ± 0.120*	4.12% ± 1.62*	2.43% ± 1.03*	1.66% ± 0.92*	1.17% ± 0.74*
1,000	2.52 ± 0.132	2.27% ± 1.43*	5.21% ± 2.18	3.12% ± 1.35	2.17% ± 1.07
2,000	2.94 ± 0.182	4.54% ± 1.68	2.96% ± 1.08	1.73% ± 1.02	1.21% ± 0.86
4,000	3.15 ± 0.78	3.20% ± 1.08	1.81% ± 0.90	1.06% ± 0.78	0.83% ± 0.60
